# Freiburg Neuropathology Case Conference: A 58-year-old Patient with an Asymptomatic Cerebellopontine Angle Mass Lesion

**DOI:** 10.1007/s00062-022-01175-w

**Published:** 2022-05-11

**Authors:** M. Schwabenland, E. A. Barvulsky, J. M. Nakagawa, M. Prinz, H. Urbach, D. Erny, C. A. Taschner

**Affiliations:** 1 Department of Neuropathology, Medical Centre—University of Freiburg, Freiburg, Germany; 2 Department of Neuroradiology, Medical Centre—University of Freiburg, Breisacherstr. 64, 79106 Freiburg, Germany; 3 Department of Neurosurgery, Medical Centre—University of Freiburg, Freiburg, Germany

**Keywords:** Vestibular schwannoma, Epidermoid cyst, Meningioma, Brain metastases, Radiologic-pathologic correlation

## Case Report

**Fig. 1:**
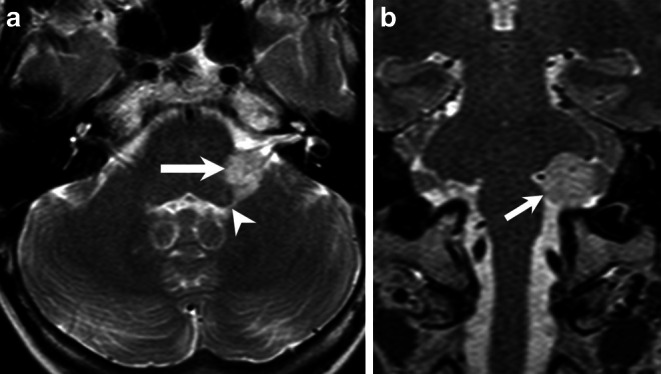
Axial T2-weighted image of the brain (**a**) shows a space-occupying lesion (*arrow*) located within the left cerebellopontine angle (CPA), encasing the cochlear nerve as well as the inferior vestibular nerve. Mind the small portion of the tumor extending into the foramen of Luschka (*arrowhead*). Coronal T2-weighted image (**b**) showed the extension of the tumor within the left CPA (*arrow*)

**Fig. 2:**
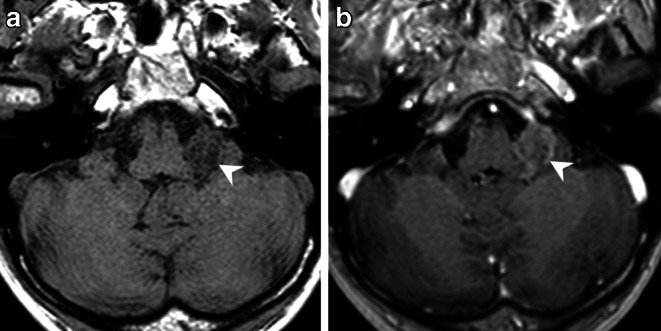
On an axial native T1-weighted image (**a**) the lesion (*arrowhead*) appears hypointense when compared to the cerebellar tissue. On an axial (**b**) T1-weighted images after administration of gadolinium the lesion shows mild partial capsular enhancement of contrast (**b**, *arrowhead*)

A 58-year-old woman presented with restless-legs syndrome and symmetrical disturbance of sensibility for both feet and the fingers of both hands. As part of an extended neurological work-up, a magnetic resonance imaging (MRI) of the head was performed. The cranial MRI revealed an incidental finding of a mass in the left cerebellopontine angle (Figs. 1 and 2). The patient had no clinical signs of swallowing difficulties, no hoarseness and the motility of the tongue was intact. The facial nerve function and hearing were not impaired. Preoperative otolaryngological evaluation confirmed intact cranial nerve (CN) function. The presenting symptoms were later attributed to a polyneuropathy.

**Fig. 3:**
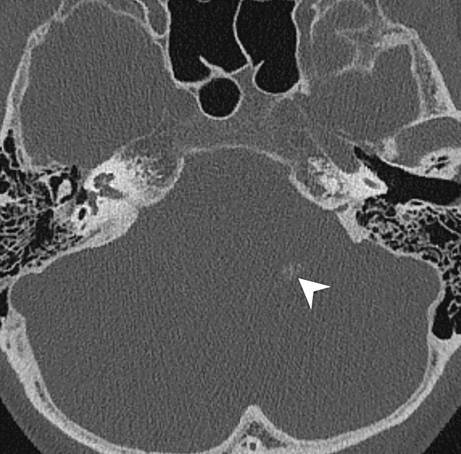
Axial CT image of the posterior fossa in bone window setting displays a sprinkled, sand-like calcification within the lesion (*arrowhead*)

Due to the size of the lesion of unknown etiology with incipient mass effect on the adjacent brainstem, surgical removal of the lesion was decided. Surgery was performed with the patient under general anesthesia in a semi-sitting position. The tumor was microsurgically resected along a retrosigmoid approach under intraoperative neurophysiological monitoring of the CN. Macroscopically the tumor was soft and of brown-grayish color and was adherent to the caudal CN, but not to CN VII/VIII. The lesion extended into the lateral aperture of Luschka. After complete resection of the tumor, the fourth ventricle and choroid plexus were clearly visualized.

Postoperatively, the patient had no neurological deficit and preserved CN function. There was an event of cardiac arrhythmia in the postoperative course, that could be sufficiently treated. The patient was discharged in a good clinical condition.

## Imaging

The cranial MRI upon admission (Fig. 1 and 2) revealed a well-circumscribed mass located within the left cerebellopontine angle (CPA). On T2-weighted images the space-occupying lesion (Fig. 1a, b) displayed encasement of the cochlear nerve as well as the inferior vestibular nerve. In addition, a small portion of the tumor extended into the foramen of Luschka (Fig. 1a). Diffusion-weighted images with a b-value = 1000 showed no signs of restricted diffusion within the lesion (not shown). On a native T1-weighted image (Fig. 2a) the lesion appeared hypointense when compared to the cerebellar tissue. On a T1-weighted image after administration of gadolinium (Fig. 2b) the lesion showed mild, partial capsular enhancement of contrast. On a CT image of the posterior fossa in bone window settings, the lesion displayed a sprinkled, sand-like calcification within the core of the lesion (Fig. 3).

## Differential Diagnosis

Tumors of the cerebellopontine angle (CPA) are quite frequent and represent about 6–10% of all intracranial tumors [1]. Based on the location within the CPA, a number of pathologies have to be considered, which are nicely summarized by the mnemonic SAME (schwannoma, arachnoid cyst/aneurysm, meningioma/metastases, ependymoma/epidermoid cyst) [2]. In this case report, we focus on those entities that were actually under consideration in our patient.

### Vestibular Schwannoma

Vestibular schwannomas (VSs) are benign extra-axial neoplasms arising from the nerve sheath of the vestibulocochlear nerve (CN VIII), usually from its vestibular division strictly following its anatomical course. They are by far the most common masses in the CPA (~ 70–80%) [3]. The peak incidence is at 50 years and patients usually present with unilateral sensorineural hearing loss (SNHL) [4]. These well-delineated tumors present as either small ovoid, homogeneously enhancing lesions within the CPA or the internal acoustic canal (IAC) or as larger lesions with a typical ice-cream-on-cone appearance extending from the CPA into the IAC [5]. Large tumors may fully occupy the CPA and cause displacement of the cranial nerves and brainstem [6].

On CT imaging widening of the osseous porus acusticus is a common feature that may reflect tumor aggressiveness. Calcifications are not present and therefore useful in differentiating it from other lesions, especially meningiomas [1, 7].

On MRI the lesion is usually isointense relative to the cerebellar parenchyma on T1WI and may rarely show small hyperintense foci in case of intralesional microhemorrhages with correlating low signal on T2*GRE/SWI. On fat-saturated heavily T2 weighted constructive interference in steady state (CISS) sequences the lesion shows a hypointense filling defect within the surrounding high signal CSF in the CPA cistern or IAC. On post-contrast T1WI, the lesion typically demonstrates avid and homogeneous enhancement. In the cystic subtypes that represent approximately 10% of all VSs, there is usually a strong marginal enhancement around a central low signal focus with correlating heterogeneous signal on T2WI. For further differentiation from other tumors, it is important to mention that VSs do not reveal any restriction on DWI [1, 4, 7, 8].

The normal audiogram confirming no SNHL as well as the mild calcifications seen on CT imaging and inhomogeneous contrast enhancement on MRI made this diagnosis less likely in our patient.

### Meningioma

Although meningiomas are most commonly located supratentorially, they represent about 10–15% of all masses in the CPA [8]. The peak incidence is at 45–55 years of age [9]. They are typically benign extra-axial dural-based and well-circumscribed masses arising from arachnoid meningothelial (“cap”) cells. Peritumoral vasogenic brain edema, which increases intracranial pressure and may cause neurological impairment, is found in more than 50% of all meningiomas [10].

On CT imaging, the mass is mostly hyperdense (70%), less commonly isodense (30%) relative to the brain parenchyma and may contain intralesional calcifications (20–25%) in a diffuse, focal or sand-like (psammomatous) manner. The adjacent bone occasionally shows hyperostosis (5%) or an irregular cortex along the inner table of the skull [11].

On MRI, the lesion is usually isointense to slightly hypointense relative to the cortex of the brain parenchyma on T1WI while it may show a variable signal intensity on T2WI depending on its texture and fibrous components. The so-called CSF cleft sign on T2WI is a typical feature seen in meningiomas (80%), which helps to distinguish extra-axial from intra-axial tumors. It shows a thin rim of hyperintense CSF between the tumor and the adjacent brain parenchyma. Apart from the intense homogeneous enhancement, meningiomas commonly reveal a dural tail sign on post-contrast imaging (60–72%), which appears to be a result of reactive thickening of the dura due to vascular congestion and edema. DWI restriction is not common but may present in the more locally aggressive atypical or malignant meningiomas [8, 11–13].

In our case, we considered meningioma a valid differential diagnosis based on its location and close proximity to the dura as well as the mild intralesional calcifications on CT imaging despite the rather inhomogeneous contrast enhancement.

### Epidermoid Cyst

Epidermoid cysts, also called pearly tumors due to their macroscopic pearl-like appearance [14], are uncommon congenital tumors arising from inclusion of ectodermal cells during neural tube closure in 3rd–5th weeks of embryogenesis. They grow very slowly along available cisternal spaces and typically become symptomatic in middle-aged patients (20–40 years) due to their mass effect on adjacent structures [15, 16]. They represent ~ 5% of all masses in the CPA [8].

On neuroimaging, epidermoid cysts show characteristic irregular and multilobulated margins. They commonly mimic or have slightly lower density/higher signal intensity than CSF, i.e. hypodense on CT (approx. 0 HU) and hyperintense in T2WI on MRI, due to the accumulation of cholesterol and keratin, breakdown products derived from desquamated epithelial cells. Very rarely, marginal calcifications or slight contrast enhancement may be seen. A key feature that helps differentiate epidermoid cysts from other lesions, especially from the otherwise indistinguishable arachnoid cysts, is the characteristic hyperintensity on DWI with ADC values equal to the brain parenchyma [1, 8, 15].

In our case, the contrast enhancement and missing diffusion restriction as well as the more advanced age made this diagnosis very unlikely.

### Brain Metastases

Metastases in the CPA disseminate through CSF infiltrating the subarachnoid space and most commonly originate from systemic tumors, such as breast or lung carcinoma, very rarely the primary tumor lies within the central nervous system (CNS). Altogether, they represent only about 0.3–0.7% of all lesions in this particular area [17]. The peak prevalence is at 65 years or older and the symptoms are related to their local mass effect [18]. The imaging characteristics may vary but are usually isointense on T1WI and T2WI and may reveal moderate to intensive contrast enhancement with or without central cystic/necrotic components [17]. DWI may show restricted diffusion in cases of high cellularity [19].

Metastases should always be considered as a differential diagnosis in a patient of advanced age with an enhancing intracranial lesion. In our case no underlying tumor was known and the patient had no B symptoms.

### Choroid Plexus Papilloma (CPP) and Choroid Plexus Carcinoma (CPCa)

Along with the atypical choroid plexus papilloma (aCPP), choroid plexus papilloma (CPP) and choroid plexus carcinoma (CPCa) represent the other two recognized subgroups of choroid plexus tumors that in about 40% of the cases arise as lobulated cauliflower-like, contrast-enhancing masses within the 4th ventricle and/or foramina of Luschka at the CPA. CPP and aCPP are indistinguishable on imaging alone while CPCa may be more heterogeneous and is more likely to invade the adjacent brain [20, 21]. On non-contrast head CT, CPP and CPPCa are isodense to hyperdense as compared with brain parenchyma. Internal calcification is present in up to 20% of cases. On MRI, CPP and CPCa are typically isointense to hyperintense on T2 and isointense to hypointense on T1. Most CPP and CPCa show robust and homogeneous enhancement on both CT and MR images [22]. Due to the strict CPA location of the underlying lesion, we initially did not consider CPP or CPCa. In addition, the pattern of contrast enhancement seemed less homogeneous than we would have expected in a CPP or CPCa. A diagnostic hint might have been the tumor part extending into the left foramen of Luschka.

## Histology and Immunohistochemistry

**Fig. 4:**
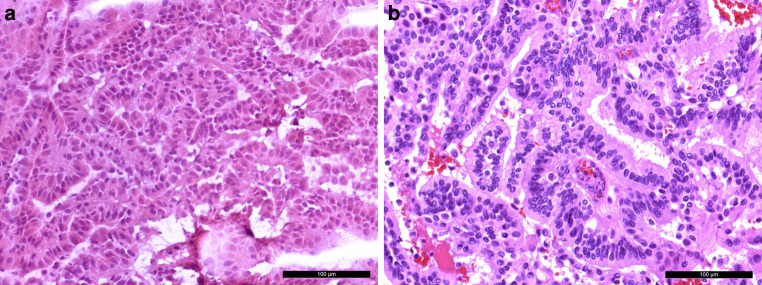
An intraoperative hematoxylin and eosin (H&E) stained cryostat section (**a**) showed a tumor with a papillary growth pattern. The H&E slide of FFPE tissue (**b**) confirmed a papillary growth pattern with a single layer of monomorphic cells. The scale bars represent 100 µm

A tissue biopsy was obtained for intraoperative neuropathological examination. A hematoxylin and eosin (H&E) stained cryostat section revealed a tumor with a papillary growth pattern (Fig. 4a). Therefore, a papillary adenoma or a metastasis of an adenocarcinoma was initially suspected. After surgical resection, further tumor tissue was fixed in formaldehyde and embedded in paraffin (FFPE).

In accordance with the intraoperative (H&E) slide, the H&E stained FFPE tissue showed a tumor with a papillary growth pattern (Fig. 4b). A single layer of monomorphic cells surrounded fibrovascular fronds. Fresh bleedings were visible. Locally, a more solid growth pattern was observed.

**Fig. 5:**
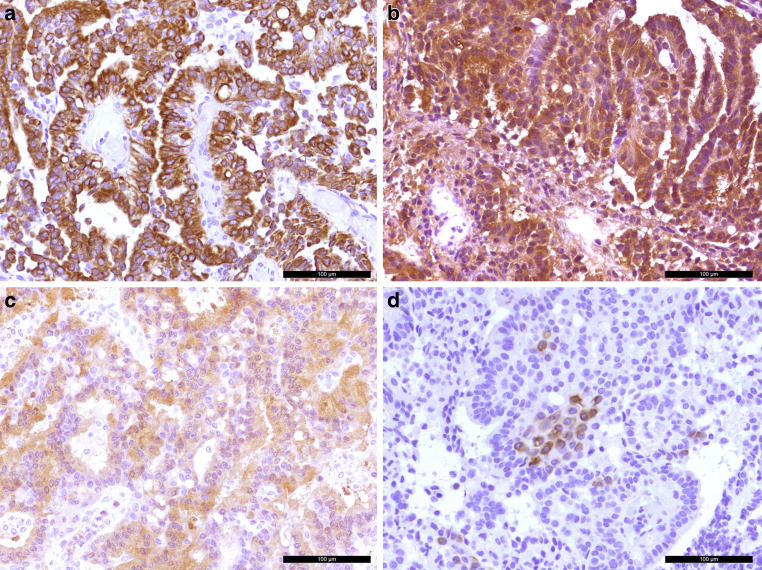
The tumors cells were positive in the immunohistochemical reaction for the pan-cytokeratin marker MNF116 (*brown*, **a**). The papilloma cells also show signal in the immunohistochemistry for transthyretin (*prealbumin*, **b**). Weak signal was observed in the immunohistochemical reaction for S100 (**c**). Tumor cells were partially labelled in the immunohistochemical reaction for cytokeratin 7 (*brown*, **d**). Hematoxylin (*blue*) was used as counterstaining in all cases (**a**–**d**). Scale bars: 100 µm

**Fig. 6:**
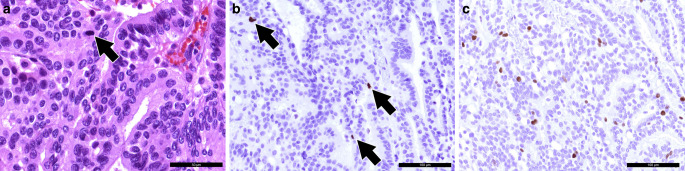
Five mitotic features were observed in 10 high-power fields (**a**, *arrow*). The scale bar represents 50 µm. Mitotic features are also visualized in the immunohistochemical reaction for phospho-HH3 (*brown*, **b**) as indicated by asterisks. Around 10% of the tumor cells were labelled in the immunohistochemical reaction for proliferation marker MIB-1 (**c**). Hematoxylin (*blue*) was used as counterstaining (**b**, **c**). Scale bar (**b**, **c**): 100 µm

The tumor cells showed positivity in the immunohistochemical reaction for the pan-cytokeratin marker MNF116 (PanCK, Fig. 5a). The cells were also strongly marked in the reaction for transthyretin (prealbumin, Fig. 5b). A weak signal was visible in the immunohistochemistry for S100 (Fig. 5c). Some tumor cells were labelled in the immunohistochemical reaction for cytokeratin 7 (CK7, Fig. 5d). The reactions for glial fibrillary astrocytic protein (GFAP), epithelial membrane antigen (EMA) and cytokeratin 20 (CK20) remained negative (not shown). Five mitotic features were observed in 10 high-power fields (HPF) (Fig. 6a). Those mitotic features could also be visualized in the immunohistochemical reaction for phospho-HH3 (Fig. 6b). The proliferation marker MIB‑1 (Ki-67) marked up to 10% of the tumor cells (Fig. 6c).

Due to the location of the tumor and the suspected clinical diagnosis, a schwannoma has been discussed as differential diagnosis; however, a biphasic growth pattern and spindle cells could not be observed in the specimen. Therefore, the histological features did not fit to a schwannoma. Moreover, the histological characteristics did not resemble those of a meningioma. EMA negativity also speaks against the presence of a meningeal tumor. The presence of a metastasis of an adenocarcinoma was discussed due to the papillary growth pattern. Nevertheless, the immunohistochemical profile including transthyretin positivity did not match an adenocarcinoma metastasis. The tumor was graded as atypical choroid plexus papilloma based on the increased mitotic activity. The criteria of a choroid plexus carcinoma such as increased nuclear pleomorphism, necrotic areas or more than 5 mitoses in 10 HPF were not fulfilled [23].

## Diagnosis

### Atypical Choroid Plexus Papilloma (WHO Grade 2)

The fifth edition of the WHO classification of central nervous system tumors defines atypical choroid plexus papilloma as a choroid plexus papilloma with an increased mitotic activity that is not fulfilling the criteria for choroid plexus carcinoma [23]. Atypical choroid plexus papillomas are graded as CNS WHO grade 2 and account for around 7% of plexus tumors [24, 25].

Choroid plexus papillomas in general are thought to arise from progenitor cells of plexus epithelium [23]. Consequently, plexus papillomas are located within the ventricular system [23]. Most of the tumors occur in children [25]. Increased intracranial pressure due to the blockage of cerebrospinal fluid drainage is a typical clinical sign of tumor entity [23]. After surgical resection, choroid plexus papillomas, CNS WHO grade 1, have a very good prognosis with a 5-year survival of more than 97% [23]. By definition, atypical choroid plexus papillomas present with an increased mitotic activity and a slightly worse overall 5‑year survival of 89% [24]. Although benign, CPPs may metastasize, mandating total surgical resection if possible [26, 27]. Rarely, CPPs can present as cerebellopontine angle (CPA) tumors from direct extension of the tumor out the foramen of Luschka or from seeding along cerebrospinal fluid pathways [22]. When a CPP presents as a CPA lesion, clinical diagnosis is not straightforward because symptoms from tumors in this area correlate more with which nerves and cerebral structures are involved, rather than the specific tumor type [22]. Chemotherapy and radiotherapy for choroid plexus tumors should be considered [28].
